# Supplementary material for: Non-canonical dihydrolipoyl transacetylase promotes chemotherapy resistance via mitochondrial tetrahydrofolate signaling
Source: Nat Commun. 2025 Oct 8;16:8932. doi: 10.1038/s41467-025-63892-3 (PMC12508156; doi:10.1038/s41467-025-63892-3)
Supplement: Supplementary file 1 — Supplementary Information [file 41467_2025_63892_MOESM1_ESM.pdf]

Non-canonical dihydrolipoyl transacetylase promotes chemotherapy resistance via mitochondrial tetrahydrofolate signaling
Hwang et al.

SUPPLEMENTARY INFORMATION

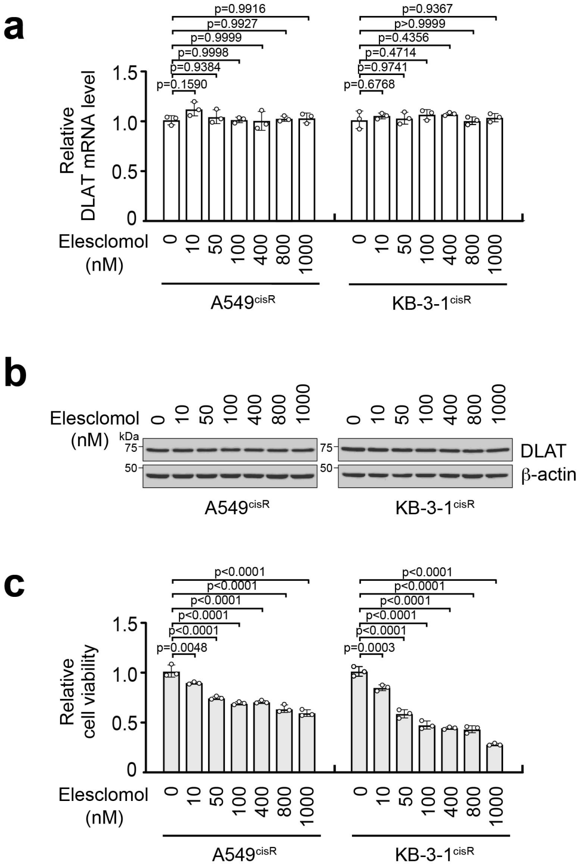

Supplementary Fig. 1: Elesclomol, a potent copper ionophore that is known to induce DLAT in prostate cancer, did not alter DLAT expression at a gene and protein level. Elesclomol was administered at increasing doses up to 1 μM for 48 h. The levels of DLAT mRNA (**a**), DLAT protein (**b**), and cell viability (**c**) are shown. Error bars represent the standard deviation (SD) and from 3 independent biological replicates for (a,c). P values were obtained by one-way ANOVA.

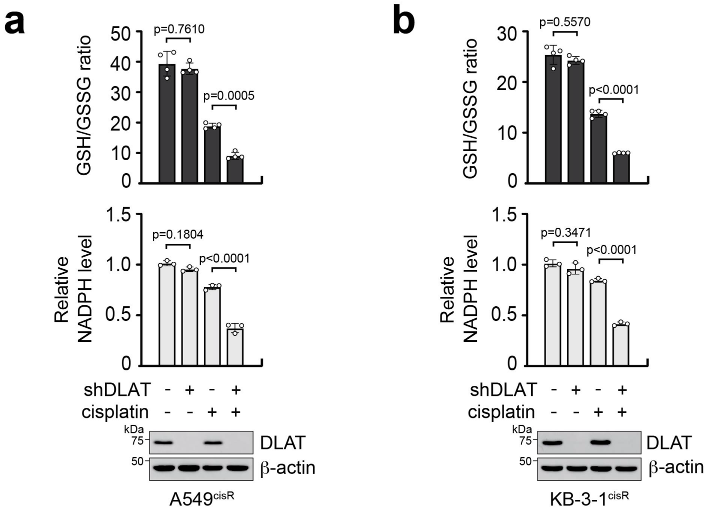

Supplementary Fig. 2: GSH/GSSG ratio and NADPH level change upon DLAT knockdown and cisplatin treatment. A549^{cisR} **(a)** and KB-3-1^{cisR} **(b)** cells were transduced with control or DLAT-targeting shRNA and treated with or without sublethal doses of cisplatin (A549^{cisR}: 2 µg/ml; KB-3-1^{cisR}: 5 µg/ml) for 24 h. The GSH/GSSG ratio and relative NADPH levels were measured using GSH/GSSG-Glo Assay and NADP/NADPH-Glo Assay, respectively. Error bars represent SD and are from 4 independent biological replicates for the upper panels of (a,b) and 3 for the lower panels of (a,b). P values were determined by one-way ANOVA.

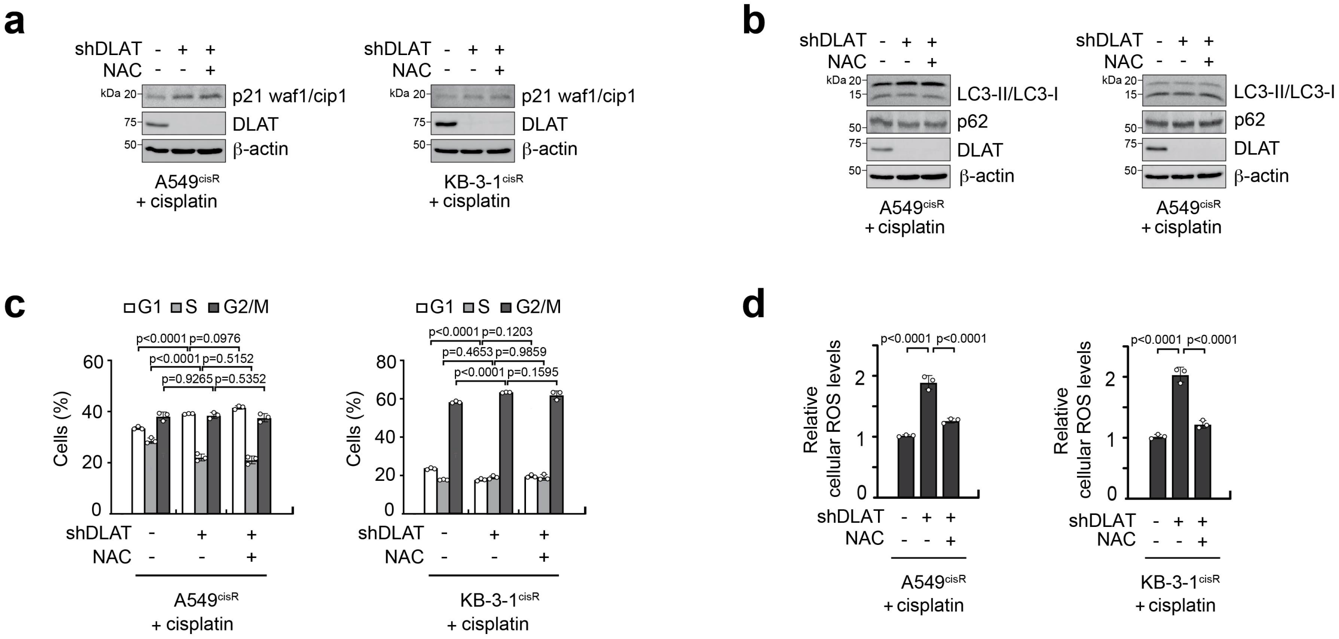

Supplementary Fig. 3: NAC treatment suppressed ROS induced by DLAT loss and cisplatin treatment but did not alter senescence, autophagy, or cell cycle progression. A549^{cisR} and KB-3-1^{cisR} cells were transduced with DLAT-targeting shRNA and treated with sublethal doses of cisplatin (A549^{cisR}: 2 µg/ml; KB-3-1^{cisR}: 5 µg/ml) for 24 h in the presence or absence of 0.5 mM antioxidant N-acetylcysteine (NAC). (**a**) Western blot analysis of p21 waf1/cip1 expression as a marker of cellular senescence. (**b**) Western blot analysis of LC3-I/II and p62 as markers of autophagy. (**c**) Cell cycle distribution was assessed by flow cytometry following propidium iodide staining. (**d**) Intracellular ROS levels were measured using the H2DCFDA probe. Error bars represent SD and are from 3 independent biological replicates for (c,d). P values were determined by one-way ANOVA.

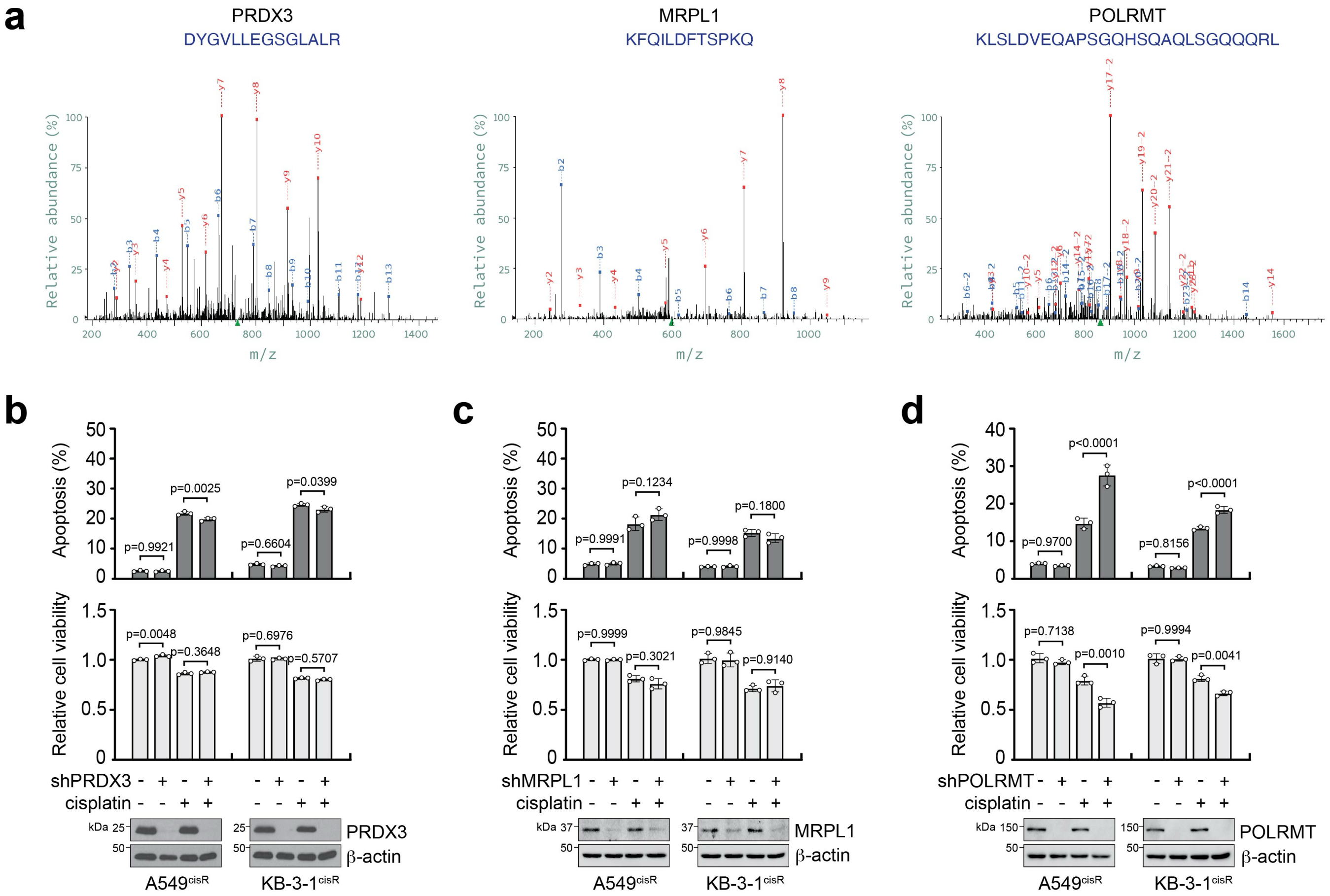

Supplementary Fig. 4: Identification of DLAT-interacting mitochondrial proteins by LC-MS/MS and evaluation of their roles in cisplatin sensitivity. (**a**) Representative MS spectra of PRDX3, MRPL1, and POLRMT peptide fragments are shown. (**b-d**) Effect of potential DLAT targets on cisplatin resistance. A549^{cisR} and KB-3-1^{cisR} cells with PRDX3 (b), MRPL1 (c), or POLRMT (d) knockdown were treated with sublethal doses of cisplatin (A549^{cisR}: 2 µg/ml; KB-3-1^{cisR}: 5 µg/ml) for 48 h. Apoptotic rates were measured by annexin V staining, and cell viability was determined using the CellTiter Glo assay. Target knockdown efficiency was confirmed by immunoblotting. Error bars represent SD and are from 3 independent biological replicates for (b-d). P values were determined using one-way ANOVA.

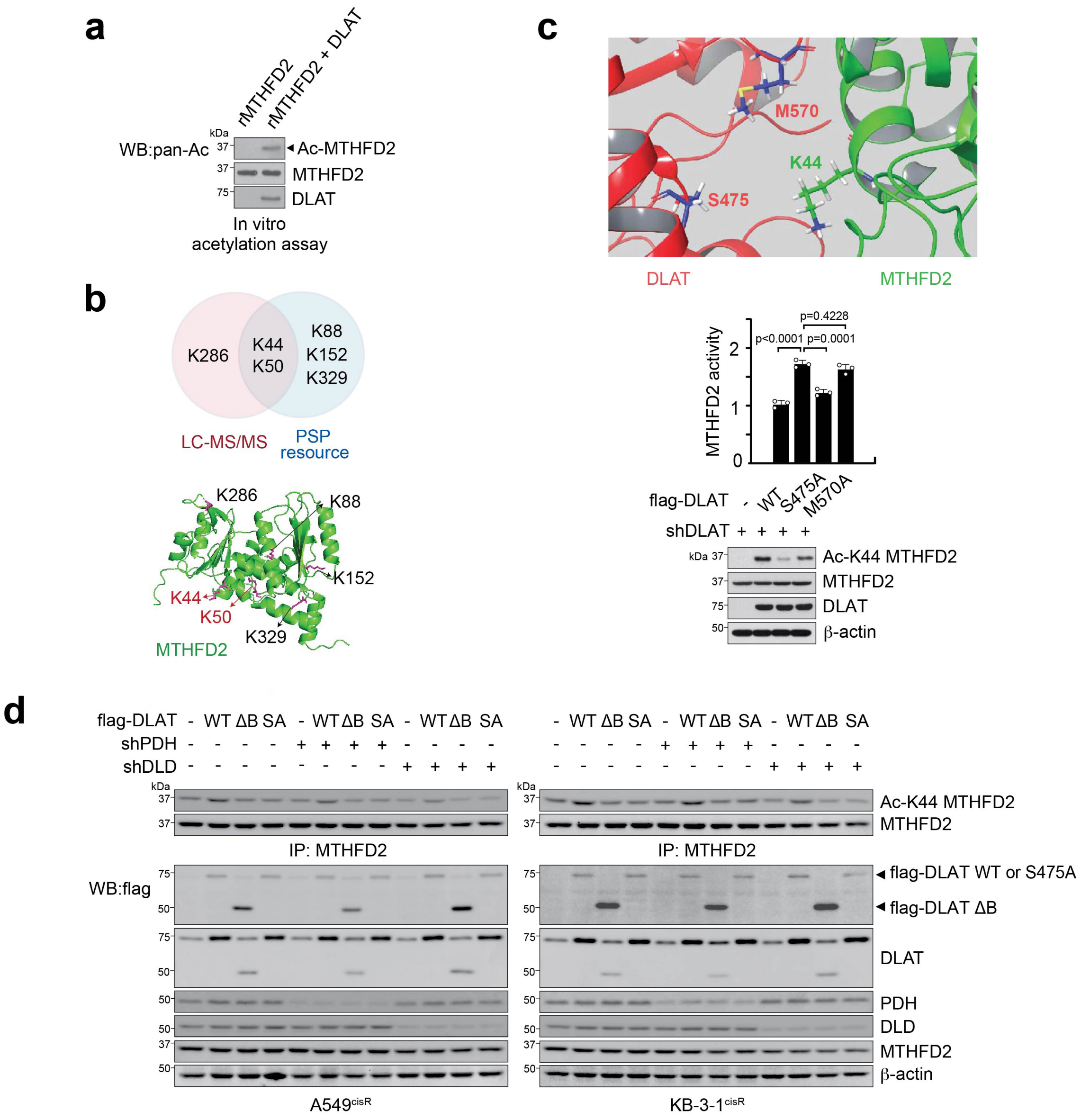

Supplementary Fig. 5: DLAT mediates MTHFD2 acetylation at lysine 44 independent of PDC. (**a**) In vitro acetylation assay using bead-bound flag-DLAT and recombinant MTHFD2 as a substrate with 10 µM acetyl-CoA. (**b**) Top: Venn diagram showing MTHFD2 acetylation sites that appeared in acetyl-proteomic analysis of (a) and PhosphoSitePlus database. Bottom: The predicted structure of human MTHFD2 (AF-P13995-F1) was obtained from the AlphaFold Protein Structure Database. The acetylation sites in MTHFD2 are indicated. (**c**) Top: Protein-protein docking of DLAT and MTHFD2 using Schrödinger (PDB:6CT0, 5TC4). The two residues in DLAT involved in MTHFD2 K44 acetylation are marked. DLAT: red, MTHFD2: green. Bottom: Effect of S475 and M570 DLAT alteration on K44 MTHFD2 acetylation and activation. DLAT S475A or M570A was rescue-expressed in DLAT knockdown cells. Error bars represent SD from three technical replicates. **(d)** Effect of PDH or DLD knockdown on DLAT-mediated MTHFD2 K44 acetylation. A549^{cisR} and KB-3-1^{cisR} cells with PDH or DLD knockdown were overexpressed with flag-DLAT variants, and acetyl-K44 MTHFD2 levels were determined by immunoblotting. Error bars represent SD and are from 3 independent biological replicates for (c). P values were determined by one-way ANOVA.

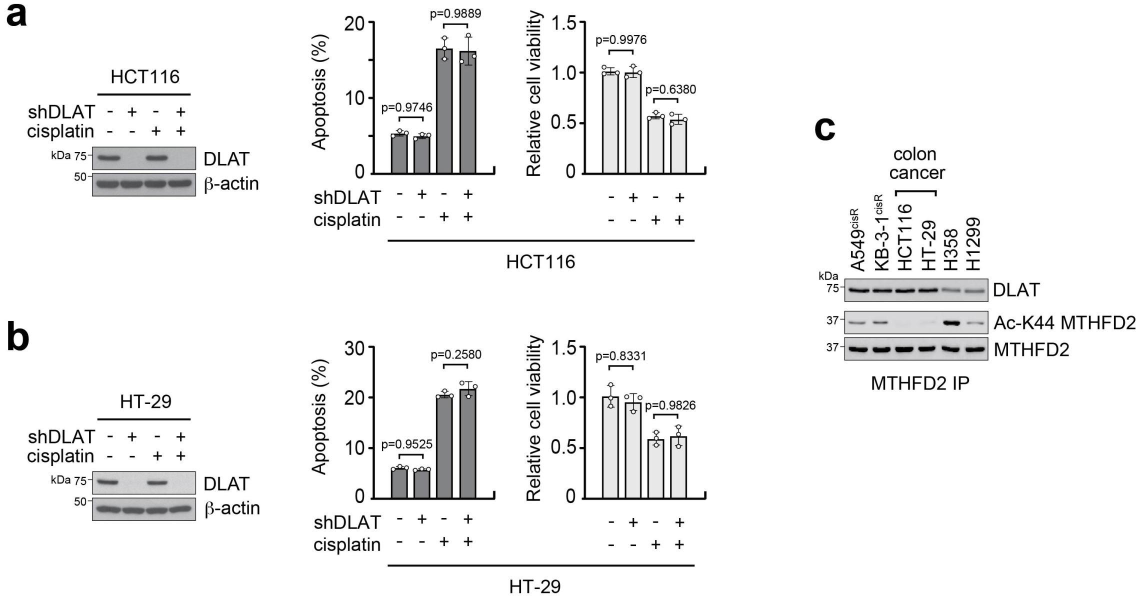

Supplementary Fig. 6: DLAT was not involved in MTHFD2 K44 acetylation and cisplatin resistance in colon cancer. **(a,b)** Effect of DLAT knockdown and cisplatin treatment on colon cancer cell viability and apoptotic cell death. Colon cancer cell lines, HCT116 (a) and HT-29 (b), were stably transduced with DLAT shRNA harboring lentivirus, followed by annexin V staining and CellTiter Glo Viability assay. Error bars represent SD and are from 3 independent biological replicates. P values were obtained by one-way ANOVA. **(c)** DLAT-mediated acetylation of MTHFD2 at K44 is observed in lung cancer cell lines, including H358 and H1299, as well as A549^{cisR} and KB-3-1^{cisR}, but not in colon cancer cell lines HCT116 and HT-29.

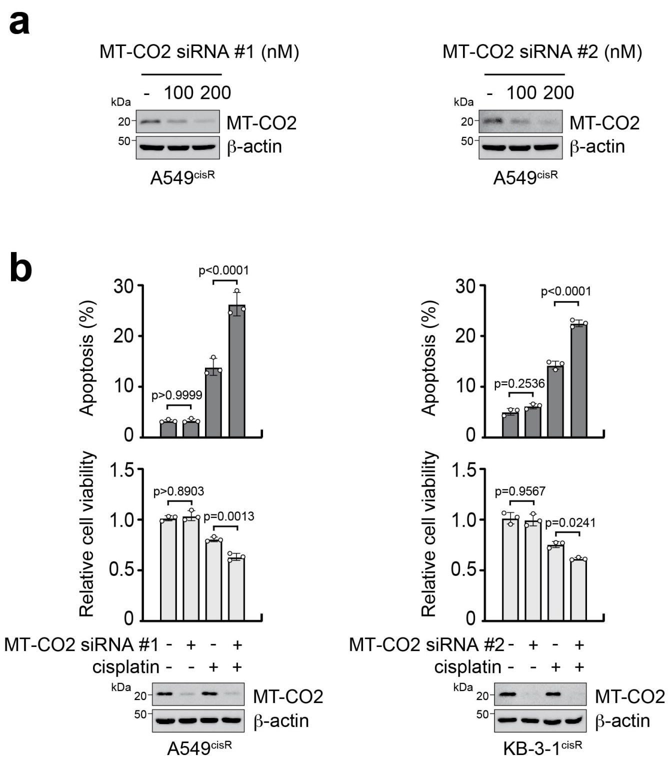

Supplementary Fig. 7: Knockdown of MT-CO2 sensitizes cancer cells to cisplatin treatment. (**a**) A549^{cisR} and KB-3-1^{cisR} cells were transfected with siRNA targeting MT-CO2 at the indicated concentrations, and the downregulation of MT-CO2 was confirmed by immunoblotting. (**b**) The effect of MT-CO2 knockdown on cisplatin sensitivity. Cells transfected with MT-CO2 siRNA were incubated with sublethal doses of cisplatin (A549^{cisR}: 2 µg/ml; KB-3-1^{cisR}: 5 µg/ml) for 48 h. Apoptosis was assessed by annexin V staining, and cell viability was determined using the CellTiter Glo assay. Error bars represent SD and are from 3 independent biological replicates for (b). P values were determined by one-way ANOVA.

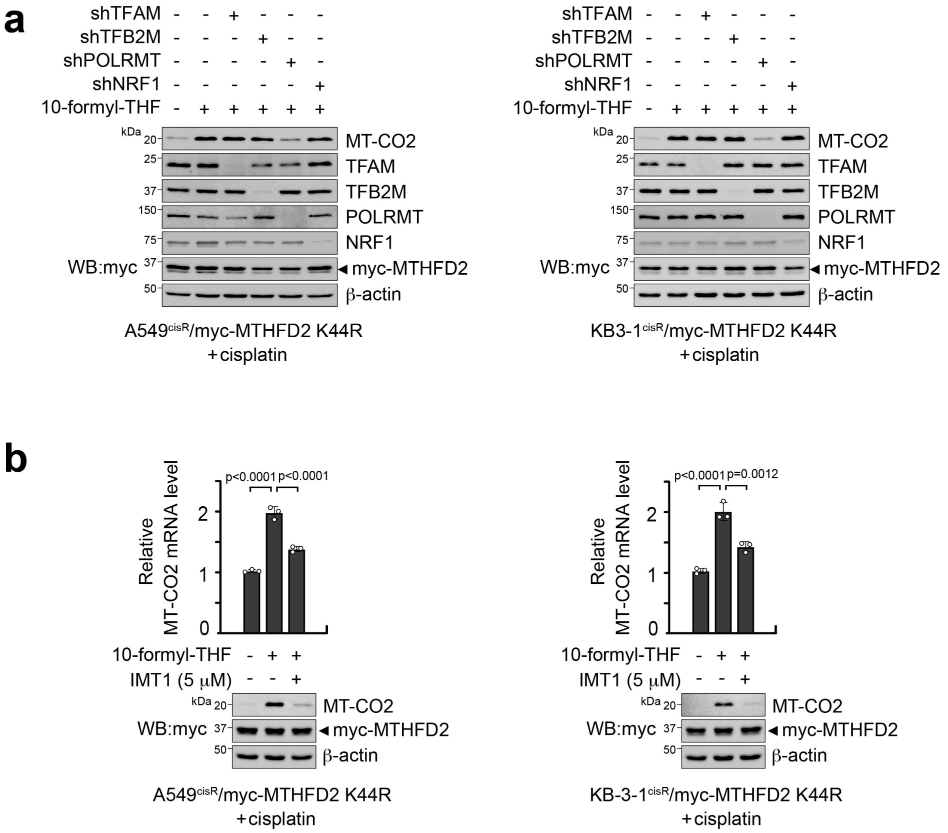

Supplementary Fig. 8: POLRMT is required for 10-formyl-THF-induced MT-CO2 expression in cisplatin-resistant cells. (**a**) A549^{cisR} and KB-3-1^{cisR} cells stably expressing myc-MTHFD2 K44R were transduced with shRNAs targeting TFAM, TFB2M, POLRMT, or NRF1, and subsequently treated with cisplatin (A549^{cisR}: 2 μg/ml, KB-3-1^{cisR}: 5 μg/ml) in the presence or absence of 10-formyl-THF (10 μM) for 24 h. The levels of MT-CO2, mitochondrial transcription factors, and myc-MTHFD2 were assessed by immunoblotting. (**b**) Effect of 10-formyl-THF and the POLRMT inhibitor IMT1 on MT-CO2 expression. Cells were treated with 10-formyl-THF (10 μM) and IMT1 (5 μM) for 24 h. MT-CO2 mRNA and protein levels were assessed by quantitative RT-PCR and immunoblotting, respectively. Error bars represent SD and are from 3 independent biological replicates for (b). P values were determined by one-way ANOVA.

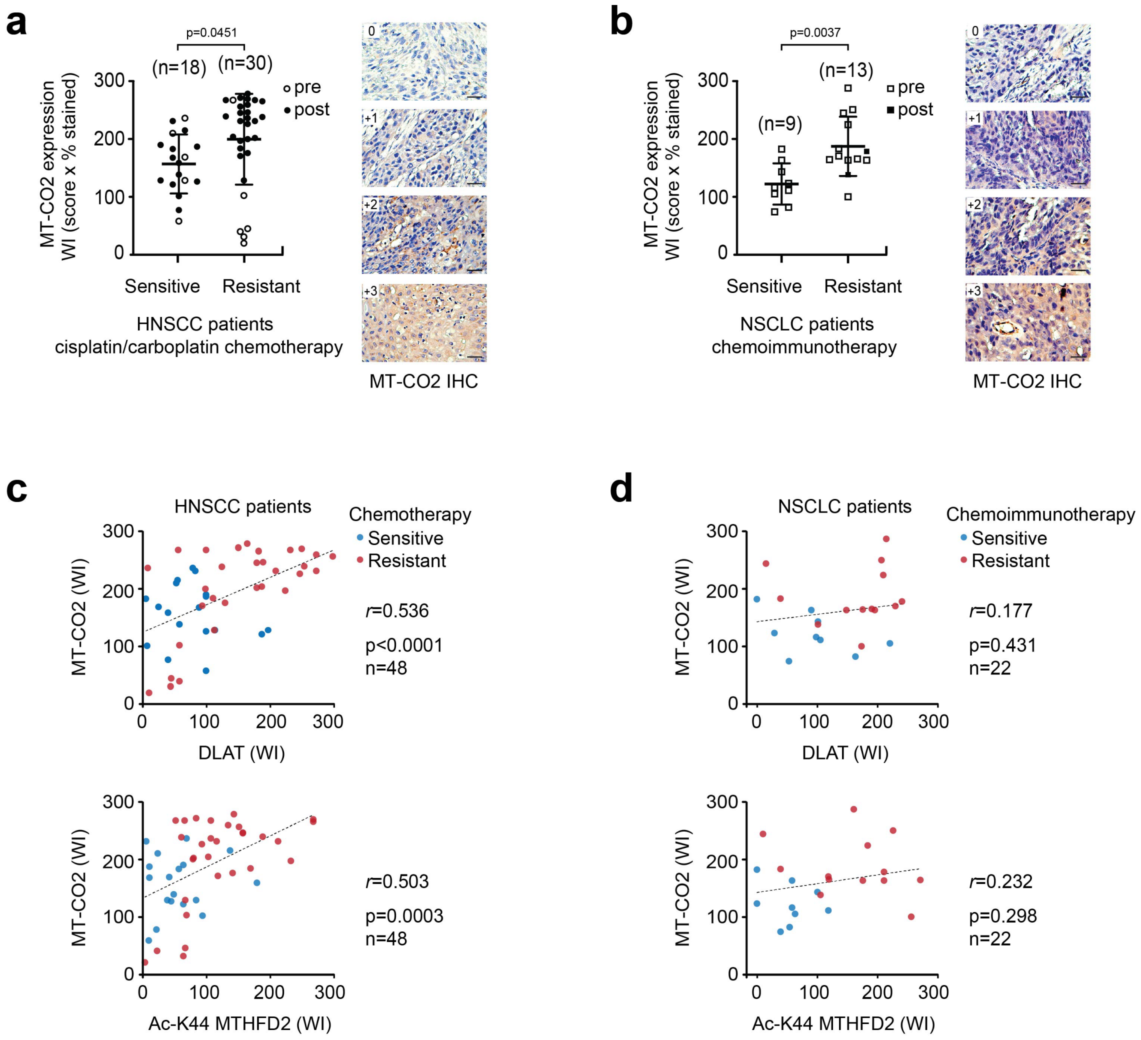

Supplementary Fig. 9: MT-CO2 correlates with poor clinical response in patients with head and neck or lung cancer receiving chemotherapy. (**a**) MT-CO2 IHC staining scores of tumors collected from HNSCC patients treated with cisplatin or carboplatin-containing regimens. Sensitive: no evidence of disease for 2 years after treatment; Resistant: disease recurred within 2 years of treatment. **(b)** MT-CO2 IHC staining scores of NSCLC patients receiving chemoimmunotherapy. Sensitive: stable disease or partial response; Resistant: progressive disease with > 20 % growth in the tumor size or spread of the tumor since the beginning of treatment. Weighted index (WI) scores were calculated by multiplying staining intensity (0~3+) by the percentage of positive staining. Four random tumor regions per image were analyzed using ImageJ version 1.54p. Representative IHC images for the score are shown. Scale bars: 20 µm. (**c,d**) Correlation analyses were performed between MT-CO2 expression and DLAT or Ac-K44 MTHFD2 levels in tumor tissues from HNSCC (c; n = 48) and NSCLC (d; n = 22) patients. Pearson correlation coefficients (r) and p-values are indicated. Error bars represent SD for (a,b). n=18 (sensitive) and n=30 (resistant) for (a), n=9 (sensitive) and n=13 (resistant) for (b), n=48 for (c), n=22 for (d). P values were obtained by unpaired two-tailed Student's t-test for (a,b) and two-tailed Pearson correlation for (c,d).

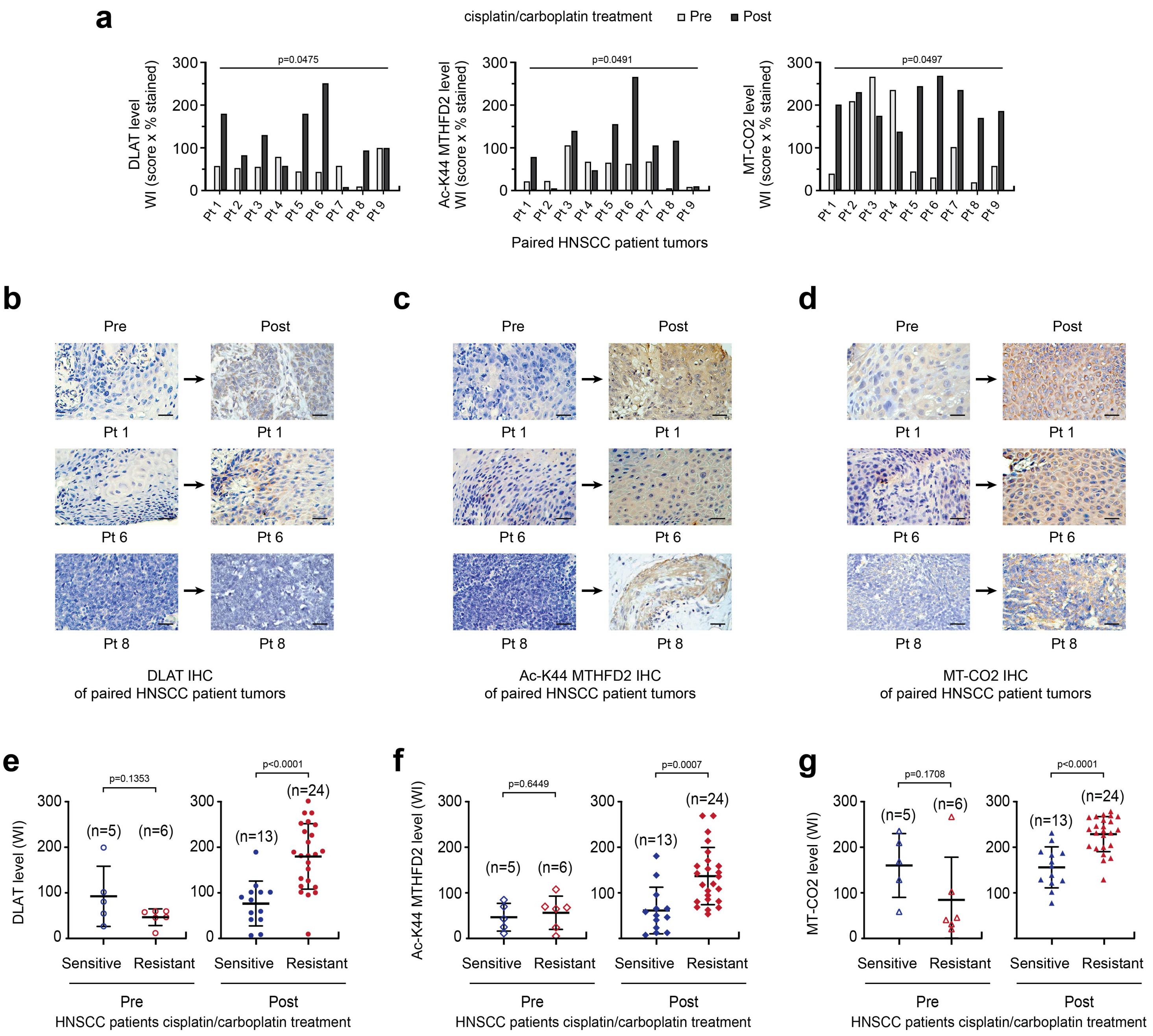

Supplementary Fig. 10: DLAT, ac-K44 MTHFD2, and MT-CO2 levels are increased in chemotherapy-treated HNSCC tumors. (**a**) IHC staining scores of DLAT, ac-K44 MTHFD2, and MT-CO2 in paired pre- and post-therapy biopsy tumor samples from HNSCC patients receiving cisplatin or carboplatin-containing regimen. (**b-d**) Representative IHC images of DLAT (b), Ac-K44 MTHFD2 (c), and MT-CO2 (d) in pre- and post-treatment paired tumors shown in (a). Scale bars: 20 µm. (**e-g**) DLAT (e), K44 MTHFD2 acetylation (f), and MT-CO2 (g) levels in tumor samples from pre-therapy biopsy and post-therapy biopsy of HNSCC patients were determined by IHC staining. Expression levels were characterized by weighted index [WI=positive staining (%) x intensity score (0~3+)]. Four random tumor regions per image were analyzed using ImageJ. Error bars represent SD for (e-g). n=5 (sensitive) and n=6 (resistant) for pre-treatment samples, and n=13 (sensitive) and n=24 (resistant) for post-treatment samples for (e-g). P values were obtained by paired (a) and unpaired two-tailed (e-g) Student's t-test.

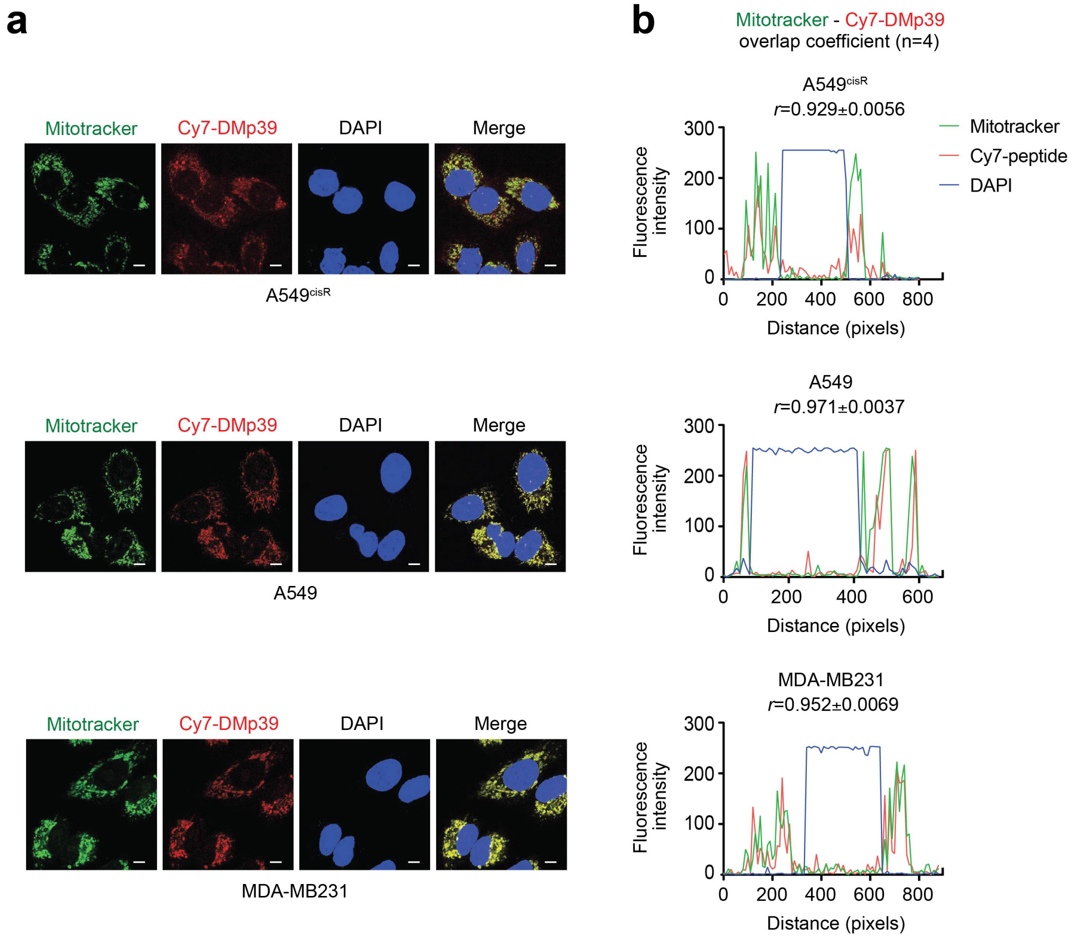

Supplementary Fig. 11: Colocalization of cy7-DMp39 with mitochondria in cancer cells. (**a**) A549^{cisR}, A549, and MDA-MB231 cells were incubated with cy7-conjugated DMp39 (cy7-DMp39) for 24 h, followed by staining with Mitotracker and DAPI. Colocalization was visualized by confocal microscopy. Scale bars: 10 µm. (**b**) Line intensity profile analysis of Mitotracker (green), cy7-DMp39 (red), and DAPI (blue) signals was performed to assess colocalization. Overlap coefficients (r) were calculated using the JACoP plugin in ImageJ.

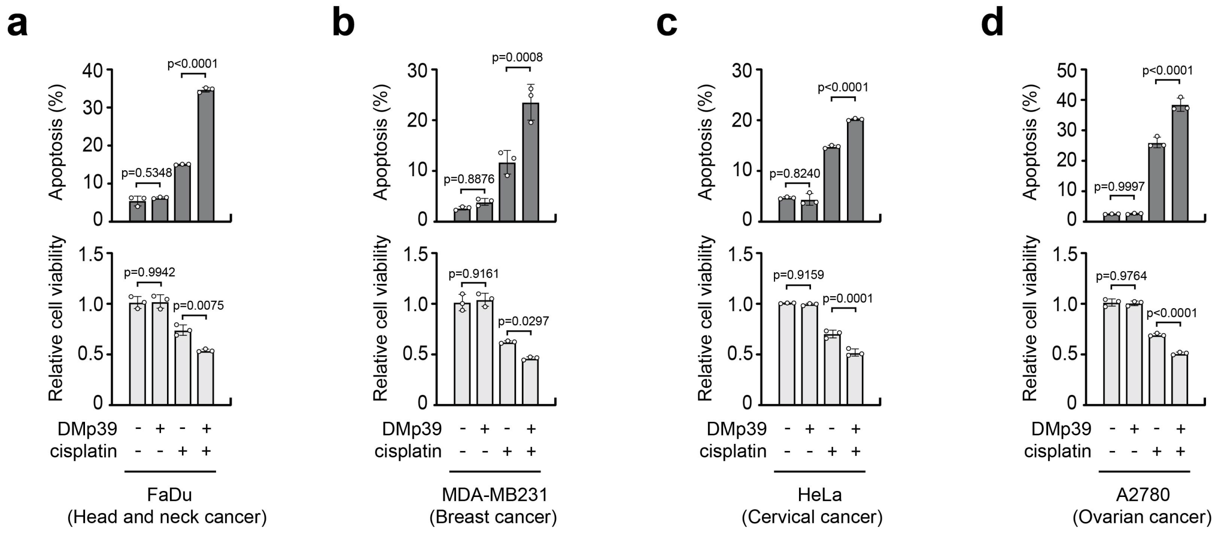

Supplementary Fig. 12: DMp39 enhances cisplatin sensitivity across multiple cancer cell lines. Cells were treated with DMp39 (20 μM) and sublethal doses of cisplatin (FaDu, MDA-MB231, HeLa: 2 μg/ml; A2780: 0.5 μg/ml) for 48 h. Apoptotic rates and cell viability were assessed by annexin V staining and CellTiter Glo Luminescent Cell Viability Assay, respectively. Error bars represent SD and are from 3 independent biological replicates for (a-d). P values were determined by one-way ANOVA.

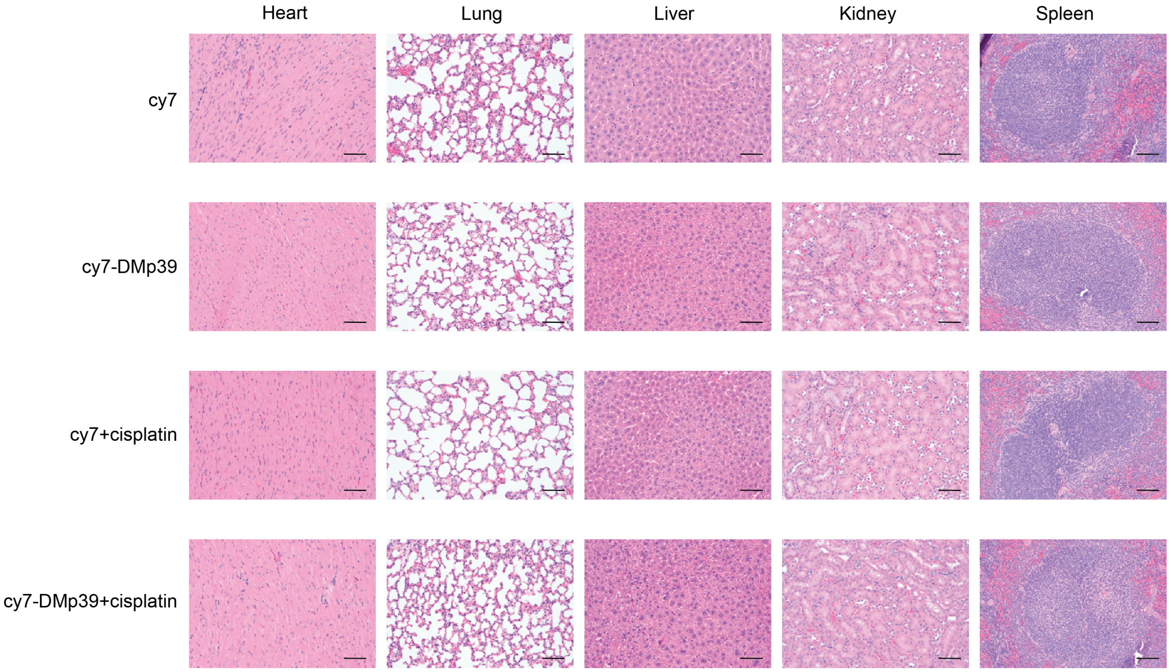

Supplementary Fig. 13: Histological analysis of hematoxylin-eosin-stained tissue sections of representative mice in cy7 control, cisplatin, cy7-DMp39, or cisplatin and cy7-DMp39 treated groups. Scale bars: 50 μm. Mice were treated with cisplatin (5 mg/kg) and DMp39 (0.1 mg/kg) twice a week for 22 days.

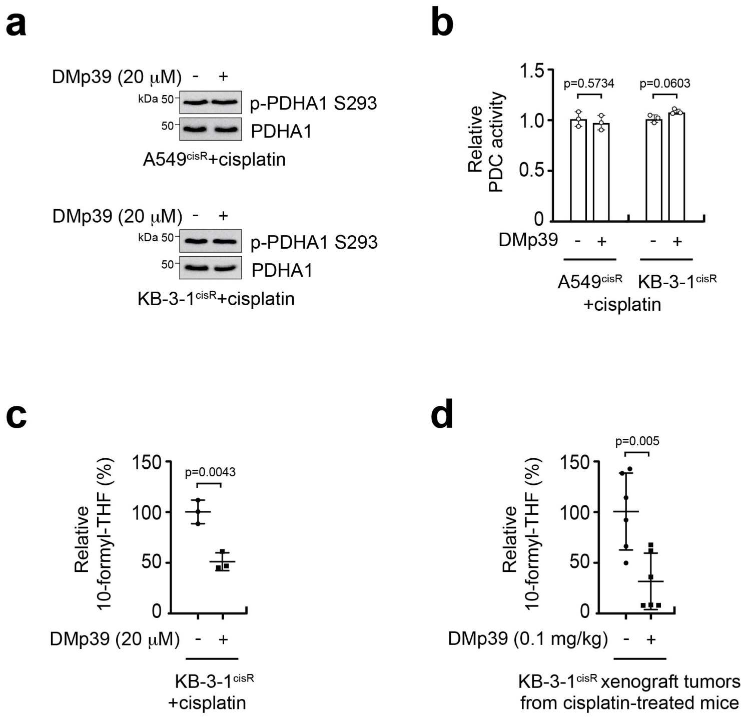

Supplementary Fig. 14: 10-formyl-THF levels *in vitro* and *in vivo* indicate that cy7-DMp39 inhibits DLAT-MTHFD2 but does not impact PDC. (**a,b**) Effect of DMp39 on PDC activity. A549^{cisR} and KB-3-1^{cisR} cells were treated with cisplatin and DMp39 (20 µM), and PDC activity was assessed by PDHA1 S293 phosphorylation (a) and colorimetric enzymatic assay (b). (**c,d**) Effect of cy7-DMp39 on 10-formyl-THF levels in cells and xenograft mice. KB-3-1^{cisR} cells were treated with cy7-DMp39 (20 µM) (c), and KB-3-1^{cisR} xenograft mice were administered cy7 or cy7-DMp39 and cisplatin as described in Figure 8f (d). Relative levels of 10-formyl-THF were quantified by LC-MS/MS. Error bars represent SD and are from 3 independent biological replicates for (b,c) and n=6 for (d). P values were determined by an unpaired two-tailed Student's t-test.
