# Supplementary material for: Non-canonical dihydrolipoyl transacetylase promotes chemotherapy resistance via mitochondrial tetrahydrofolate signaling
Source: Nat Commun. 2025 Oct 8;16:8932. doi: 10.1038/s41467-025-63892-3 (PMC12508156; doi:10.1038/s41467-025-63892-3)
Supplement: Supplementary file 2 — Reporting Summary [file 41467_2025_63892_MOESM2_ESM.pdf]

nature portfolio

Corresponding author(s): Sumin Kang

Last updated by author(s): Aug 06, 2025

Reporting Summary

Nature Portfolio wishes to improve the reproducibility of the work that we publish. This form provides structure for consistency and transparency in reporting. For further information on Nature Portfolio policies, see our Editorial Policies and the Editorial Policy Checklist.

Statistics

For all statistical analyses, confirm that the following items are present in the figure legend, table legend, main text, or Methods section.

n/a	Confirmed	
☐	☒	The exact sample size (*n*) for each experimental group/condition, given as a discrete number and unit of measurement
☐	☒	A statement on whether measurements were taken from distinct samples or whether the same sample was measured repeatedly
☐	☒	The statistical test(s) used AND whether they are one- or two-sided<br>*Only common tests should be described solely by name; describe more complex techniques in the Methods section.*
☒	☐	A description of all covariates tested
☒	☐	A description of any assumptions or corrections, such as tests of normality and adjustment for multiple comparisons
☐	☒	A full description of the statistical parameters including central tendency (e.g. means) or other basic estimates (e.g. regression coefficient) AND variation (e.g. standard deviation) or associated estimates of uncertainty (e.g. confidence intervals)
☐	☒	For null hypothesis testing, the test statistic (e.g. *F*, *t*, *r*) with confidence intervals, effect sizes, degrees of freedom and *P* value noted<br>*Give P values as exact values whenever suitable.*
☒	☐	For Bayesian analysis, information on the choice of priors and Markov chain Monte Carlo settings
☒	☐	For hierarchical and complex designs, identification of the appropriate level for tests and full reporting of outcomes
☐	☒	Estimates of effect sizes (e.g. Cohen's *d*, Pearson's *r*), indicating how they were calculated

Our web collection on statistics for biologists contains articles on many of the points above.

Software and code

Policy information about availability of computer code

Data collection	Data collection was performed using the following software:<br>BDBD FACSDiva Software v9.0 (BD Biosciences) for flow cytometry; ImageQuant 800 (Cytiva) for Western blot image acquisition; IVIS Imaging System (Perkin Elmer) for live animal imaging and monitoring; Leica SP8 confocal microscope (Leica Microsystems) for immunofluorescence image capture; Agilent ChemStation (Agilent Technologies) for LC-MS chromatographic data acquisition
Data analysis	Data analysis was performed using:<br>GraphPad Prism v10.0 (GraphPad Software) for statistical analysis and graph generation; FlowJo v10.9 (BD Biosciences) for flow cytometry analysis; ImageJ (NIH) for colony formation and image quantification; OpenLAB CDS ChemStation Edition (Rev C.01.10 (201)), Agilent) for LC-MS peak quantification; NNo custom scripts oror algorithms were used iin this study.

For manuscripts utilizing custom algorithms or software that are central to the research but not yet described in published literature, software must be made available to editors and reviewers. We strongly encourage code deposition in a community repository (e.g. GitHub). See the Nature Portfolio guidelines for submitting code & software for further information.

Data

Policy information about availability of data

All manuscripts must include a data availability statement. This statement should provide the following information, where applicable:

- Accession codes, unique identifiers, or web links for publicly available datasets
- A description of any restrictions on data availability
- For clinical datasets or third party data, please ensure that the statement adheres to our policy

The proteomics data that identified interaction partners in this study have been deposited in the ProteomeXchange with identifier PXD064757. Publicly available datasets from https://kmplot.com/analysis/ and https://www.phosphosite.org/homeAction.action were also used in this study. Source data are provided with this paper.

Research involving human participants, their data, or biological material

Policy information about studies with human participants or human data. See also policy information about sex, gender (identity/presentation), and sexual orientation and race, ethnicity and racism.

Reporting on sex and gender	Sex and gender information of patients was not available for the de-identified tumor samples used in this study.
Reporting on race, ethnicity, or other socially relevant groupings	Race and ethnicity data were not collected for the tumor samples analyzed.
Population characteristics	Tumor specimens were derived from patients with histologically confirmed HNSCC or NSCLC. No other covariate-level population characteristics were available.
Recruitment	Tumor samples were obtained through approved IRB protocols at Emory University.
Ethics oversight	This study was approved by the Emory University Institutional Review Board (IRB# IRB00003208, IRB# IRB00098377).

Note that full information on the approval of the study protocol must also be provided in the manuscript.

Field-specific reporting

Please select the one below that is the best fit for your research. If you are not sure, read the appropriate sections before making your selection.

[x] Life sciences [ ] Behavioural & social sciences [ ] Ecological, evolutionary & environmental sciences

For a reference copy of the document with all sections, see nature.com/documents/nr-reporting-summary-flat.pdf

Life sciences study design

All studies must disclose on these points even when the disclosure is negative.

Sample size	No statistical methods were used to pre-determine sample size. For in vivo experiments, a sample size of n=6-8 was used per experimental group. Sample sizes were based on previous studies (Cancer Cell 2018; PMID: 30033091; Nature Communications 2021; PMID: 34400618) and are commonly accepted in the field. For in vitro experiments, three biological replicates were included for most experiments.
Data exclusions	No data were excluded from the analyses.
Replication	Mechanistic studies were performed using three independent biological replicates. For animal studies, 8 mice per group were used in Fig. 1g and 1h, and 6 mice per group were used in Fig. 8h, 8i, 8k, and 8l. All attempts at replication were successful for these experiments.
Randomization	To minimize potential bias, equal numbers of animals (in vivo) or cells (in vitro) were randomly assigned to experimental groups.
Blinding	Investigators were not blinded to group allocation during the experiments, as the outcomes were based on objective, non-subjective measurements. To minimize potential bias, experiments were designed such that multiple samples were processed and analyzed side-by-side under consistent conditions. Appropriate controls were included in every study to ensure reliability and reproducibility.

Reporting for specific materials, systems and methods

We require information from authors about some types of materials, experimental systems and methods used in many studies. Here, indicate whether each material, system or method listed is relevant to your study. If you are not sure if a list item applies to your research, read the appropriate section before selecting a response.

Materials & experimental systems

n/a	Involved in the study
	[x] Antibodies
	[x] Eukaryotic cell lines
[x]	Palaeontology and archaeology
	[x] Animals and other organisms
[x]	Clinical data
[x]	Dual use research of concern
[x]	Plants

Methods

n/a	Involved in the study
[x]	ChIP-seq
	[x] Flow cytometry
[x]	MRI-based neuroimaging

Antibodies

Antibodies used

The following primary antibodies were used in this study:
Novus Biologicals: Anti-pyruvate dehydrogenase E2/DLAT (Cat# NBP2-34065; for IHC, 1:500 dilution), Anti-MT-CO2 (Cat# NBP2-94364; for IHC, 1:200 dilution); Santa Cruz Biotechnology: Anti-PDC-E2 (DLAT) (Cat# sc-271534, clone B-2; for immunoblotting, 1:2000 dilution), Anti-PDH1-E1! (E1) (Cat# sc-377092, clone D-6, 1:1000 dilution)), Anti-!-tubulin (Cat# sc-23948, clone B-5-1-2, 1:2000 dilution), Anti-Bax (Cat# sc-493, 1:500 dilution), Anti-Tom40 (Cat# sc-11414, clone H-300, 1:500 dilution), Anti-p21 (Cat# sc-397, clone C-19, 1:500 dilution), Anti-POLRMT/MtRPOL (Cat# sc-365082, clone B-1, 1:500 dilution); Cell Signaling Technology: Anti-MTHFD2 (Cat# 41377, clone D8W9U, 1:1000 dilution), Anti-myc-Tag (Cat# 2278, clone 71D10, 1:1000 dilution), Anti-phospho-Histone H2AX (Ser139) (Cat# 9718, clone 20E3, 1:200 dilution), Anti-phospho-53BP1 (Ser1778) (Cat# 2675, 1:200 dilution), Anti-COX1/MT-CO1 (Cat# 62101, 1:500 dilution), Anti-COX2/MT-CO2 (Cat# 31219, 1:500 dilution), Anti-COX IV (Cat# 4850, clone 3E11, 1:1000 dilution), Anti-acetyl-lysine (Cat# 9441, 1:500 dilution), Anti-Bcl-xL (Cat# 2762, 1:500 dilution), Anti-Bcl2 (Cat# 15071, 1:500 dilution), Anti-Mcl-1 (Cat# 39224, clone D5V5L, 1:500 dilution), Anti-Bad (Cat# 9268, clone 11E3, 1:500 dilution), Anti-Bim (Cat# 2933, clone C34C5, 1:500 dilution), Anti-PARP (Cat# 9542, 1:500 dilution), Anti-Histone H3 (Cat# 4499, clone D1H2, 1:500 dilution), Anti-LC3A/B-I/II (Cat# 4108, 1:500 dilution), Anti-p62 (Cat# 5114, 1:500 dilution); Sigma-Aldrich: Anti-"-actin (Cat# A1978, clone AC-15, 1:4000 dilution), Anti-FLAG (Cat# F7425, 1:1000 dilution), Anti-GST (Cat# G1160, clone GST-2, 1:2000 dilution); Abcam: Anti-PRDX3 (Cat# ab128953, clone EPR8115, 1:1000 dilution), Anti-phospho-PDHA1 (Ser293) (Cat# ab177461, clone EPR12200, 1:1000 dilution), Anti-cisplatin-modified DNA (Cat# ab103261, clone CP9/19, 1:200 dilution), Anti-Ki-67 (Cat# ab92742, clone EPR3610, 1:200 dilution); ProteinTech: Anti-DLD (E3) (Cat# 16431-1-AP, 1:1000 dilution); Abnova: Anti-MRPL1 (Cat# H00065008-M02, 1:1000 dilution); Invitrogen: Anti-mouse IgG Alexa Fluor 488 (Cat# A11001, 1:200 dilution), Anti-rabbit IgG Alexa Fluor 568 (Cat# A21069, 1:200 dilution); PTM BIO: Custom anti-acetyl-K44 MTHFD2 antibody (1:500 dilution).

Secondary antibodies used in this study include:
Invitrogen: Anti-rabbit IgG (H+L) secondary antibody–HRP (Invitrogen, Cat# 31460, 1:2000 dilution), Anti-mouse IgG (H+L) secondary antibody–HRP (Invitrogen, Cat# 31430, 1:2000 dilution)

Validation

All commercial antibodies used in this study have been validated by the respective manufacturers for the species and applications (e.g., Western blot, immunohistochemistry) employed in our experiments. Manufacturer validation details are available on the vendor websites and were taken into account during antibody selection. In addition, antibody specificity was further confirmed in this study through appropriate internal controls. For example, the anti-DLAT and anti-MTHFD2 antibodies were validated by knockdown and overexpression experiments, showing specific detection of target proteins. The custom anti-acetyl-K44 MTHFD2 antibody was generated by PTM BIO using a synthetic peptide containing acetylated lysine at position 44 of human MTHFD2. The antibody was validated by DLAT overexpression (which increased MTHFD2 acetylation), and by MTHFD2 knockdown (which eliminated the signal), confirming that it specifically detects acetylated K44 on MTHFD2. Additional validation using a peptide competition assay is shown in Fig. 8c.

Eukaryotic cell lines

Policy information about cell lines and Sex and Gender in Research

Cell line source(s)

The following human cancer cell lines were used in this study: A549, H1299, A2780, HCT116, HT-29, HeLa, 293T, MDA-MB231, FaDu, PCI-37B, and KB-3-1. Cisplatin-resistant variants (e.g., A549cisR, KB-3-1cisR) were generated in-house by chronic exposure to cisplatin. A549, H1299, HeLa, 293T, MDA-MB231, and KB-3-1 cell lines were obtained from ATCC, while HCT116, HT-29, FaDu, and PCI-37B were obtained from collaborators or established laboratory stocks. All cell lines used are of human origin.

Authentication

All cell lines were authenticated by STR profiling.

Mycoplasma contamination

All cell lines were tested negative for mycoplasma contamination.

Commonly misidentified lines
(See ICLAC register)

No commonly misidentified cell lines were used in this study.

Animals and other research organisms

Policy information about studies involving animals; ARRIVE guidelines recommended for reporting animal research, and Sex and Gender in Research

Laboratory animals

All animal studies were conducted using Nude mice (Hsd:Athymic Nude-Foxn1nu, female, 6-week-old, Envigo). Mice were housed under specific pathogen-free conditions with controlled temperature and humidity.

Wild animals	Wild animals were not used.
Reporting on sex	Only female mice were used iin this study. The study did not include sex-based comparisons, asas the focus was not onon sex-specific effects. Justification for using female mice was based onon previous publications and standard practice iin the field.
Field-collected samples	This study did not involve samples collected from the field.
Ethics oversight	All animal procedures were reviewed and approved byby the Emory University Institutional Animal Care and Use Committee (IACUC).

Note that full information on the approval of the study protocol must also be provided in the manuscript.

Plants

Seed stocks	This study did not involve the use ofof any plant materials oror seed stocks.
Novel plant genotypes	No novel plant genotypes were used or generated in this study.
Authentication	Not applicable. NoNo plant-based materials oror genotypes were used.

Flow Cytometry

Plots

Confirm that:

- [ ] The axis labels state the marker and fluorochrome used (e.g. CD4-FITC).
- [ ] The axis scales are clearly visible. Include numbers along axes only for bottom left plot of group (a 'group' is an analysis of identical markers).
- [ ] All plots are contour plots with outliers or pseudocolor plots.
- [x] A numerical value for number of cells or percentage (with statistics) is provided.

Methodology

Sample preparation	Cells were harvested, washed with PBS, and stained with Annexin V–FITC and propidium iodide (PI) using the FITC Annexin V Apoptosis Detection Kit (BD Biosciences), following the manufacturer's protocol. For cell cycle analysis, cells were fixed with 70% ethanol and stained with PI/RNase solution.
Instrument	Flow cytometry data were collected using a instrument (BD Symphony A3) and BBD FACSDiva Software v9.0.
Software	Data were analyzed using FlowJo software (v10.9, BBD Biosciences).
Cell population abundance	For apoptosis analysis, cells were stained with Annexin V–FITC and propidium iodide (PI), and the percentage ofof apoptotic cells was calculated based onon the total number ofof events. Data are presented asas bar graphs showing mean ± SDSD from three independent experiments.<br>For cell cycle analysis, cells were fixed with 70% ethanol and stained with PI/RNase solution. The distribution ofof cells across G0/G1, S,S, and G2/M phases was quantified using FlowJo's cell cycle analysis platform.
Gating strategy	For cell cycle analysis, debris and doublets were excluded using FSC/SSC and FSC-H vsvs FSC-A gating. Gating was based onon DNA content, and phases were defined using FlowJo. For apoptosis analysis, gating thresholds were determined using unstained, Annexin V-only, and PI-only controls.

- [ ] Tick this box to confirm that a figure exemplifying the gating strategy is provided in the Supplementary Information.
